# A giant cystic echinococcosis of the lung in East Turkey

**DOI:** 10.1016/j.amsu.2018.10.009

**Published:** 2018-10-12

**Authors:** Tuba Apaydın, Cem Başaran

**Affiliations:** a Thoracic Surgery Unit, Bitlis State Hospital, Beş Minare District, Selahattin Eyyübi Street. Nu:160, 13000, Bitlis, Turkey; b Anesthesiology Unit, Antalya Gazipaşa State Hospital, Cumhuriyet District, Hastane Street, 07900, Gazipaşa, Antalya, Turkey

**Keywords:** Hydatid cyst, Echinococcosis, Lung, Case report

## Abstract

**Introduction:**

Hydatid cyst disease is a zoonosis provoked by Echinoccocus.

**Presentation of case:**

17 year old male applied to our clinic with complaints of fatigue, lassitude, right chest pain and spitting of watery expectoration proceeding in 6 months. Computed tomographic scan indicated a 130*110 mm smooth contoured cavitary lesion located in the right middle lobe of the lung. Treatment with cystotomy and capitonnage successfully. Histopathologic examination confirmed hydatid cyst. The patient recovered from all his complaints postoperatively and he was discharged from the hospital in 7 days. Albendazole was implemented for 3 months postoperatively. He was observed to be healthy in his three month follow-up visit.

**Discussion:**

Pulmonary hydatid cysts are generally treated with surgery. Cystotomy and capitonnage, pericystectomy and enucleation are the commonly used surgical techniques.

**Conclusions:**

Surgery is the treatment regimen for pulmonary hydatid cysts and antihelmintic therapy is adviced to eliminate recurrences postoperatively.

## Highlights

• Hydatid disease is an endemic disease provoked by Echinococcus granulosus.
• Preoperative diagnosis of hydatid cyst is assured with clinical, radiological and serological outcomes.
• Cystotomy and capitonnage, enucleation and pericystectomy are the commonly used surgical techniques.

## 1 Introduction

Cystic echinococcosis is a zoonosis resulting with significant morbidity and mortality in the world especially in east Turkey [1]. Cestodes of echinococcus granulosus and echinoccocus alveolaris are the most common types causing infection in humans. Surgery is the treatment regimen and antihelmintic therapy is adviced to eliminate recurrences postoperatively. Here we present a 17 year old male with a giant hydatid cyst of the lung treated with thoracotomy and cystotomy-capitonnage for the cyst. This report is prepared compatible with the SCARE criteria.

## 2 Presentation of case

**Fig. 1:**
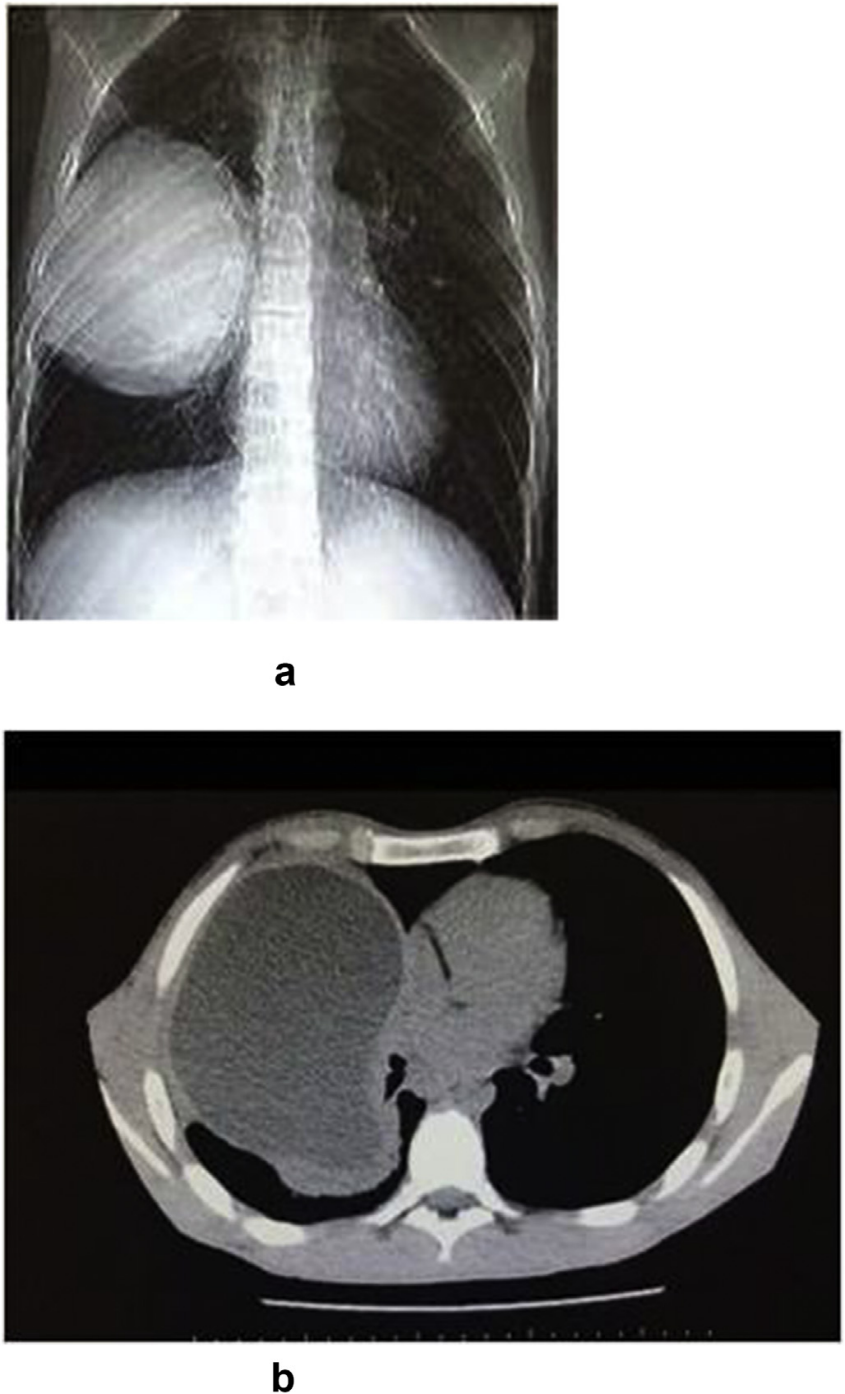
(A) Preoperative view of the Chest X-Ray. (B) Preoperative view of Thorax CT.

**Fig. 2:**
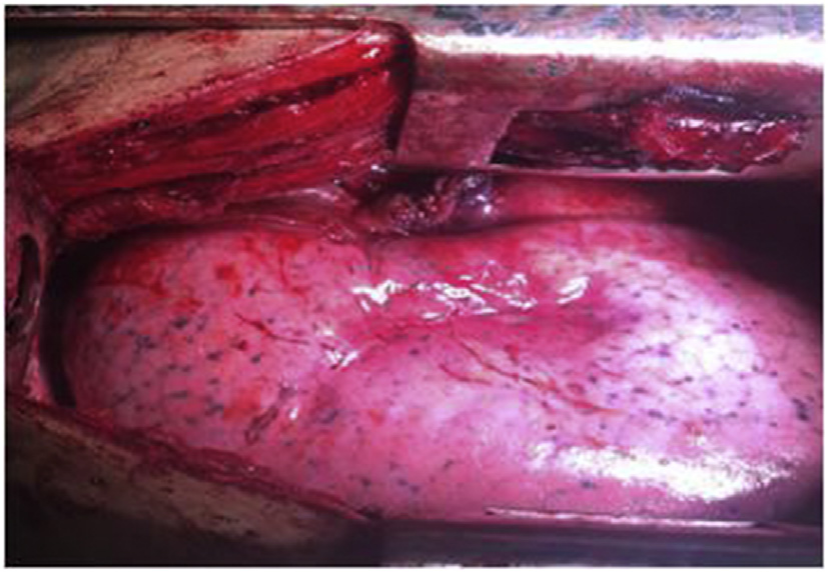
Postoperative view of the lung.

**Fig. 3:**
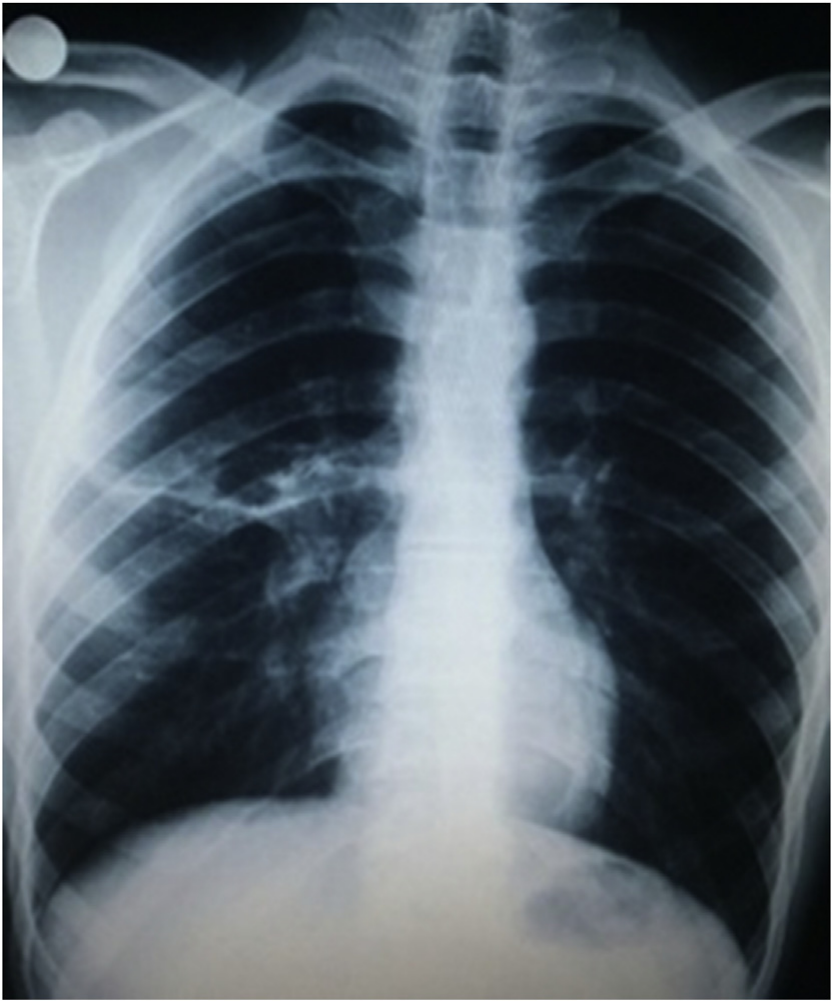
Postoperative view of the Chest X-Ray.

17 year old male applied to our clinic with fatigue, lassitude, right chest pain and spitting of watery expectoration proceeding in 6 months. He was inhabiting in a pastoral area of east Turkey where husbandry and contact with cats&dogs is common. Vital signs were normal and breath sounds were decreased in the upper and middle part of right chest in physical examination. In laboratory examination, hemoglobin was 11.8 gr/dl, white blood cell count was 15.390 (per mm3) (neutrophils 88%, eosinophils 0.5%, lymphocytes 4.9%), platelet count was 490.000 (per mm^³^). Serum biochemical analysis confirmed normal values. Serum total immunoglobulin E level wasn't analysed in our laboratory. In Chest X-Ray, a giant cystic lesion was detected in the right middle lobe of the lung (Fig. 1a). In Thorax CT, 130*110 mm well contoured lobulated cavitary lesion was detected in the right middle lobe of the lung (Fig. 1b). Also 50*30 mm Grade II cyst hydatid was detected in the right lobe of the liver in abdominal CT. After a right posterolateral thoracotomy from 5. Intercostal area, in the exploration right upper lobe was adherent to the thoracic wall from apical and posterolateral region. Adherences were separated with blunt and sharp dissection. Some of the cystic fluid was drained with the needle aspiration and cystotomy was implemented. Ingredients of the cyst and germinative membrane were aspirated. Cavity was washed with iodine solution and hypertonic saline solution. Bronchial openings were controlled and renovated. Capitonnage was applied to the cavity (Fig. 2). Chest tubes were inserted to the thoracic cavity and patient was extubated in the operation room. Basal chest tube was fetched in postoperative 2. day, apical chest tube was fetched in postoperative 5. day. In postoperative 7. day, patient was discharged from the clinic with complete recovery from the complaints. Histopathologic survey of the cyst certified the diagnosis. Antihelminthic therapy (albendazole, 10 mg/kg) was implemented per day for 3 months postoperatively without hepatotoxicity. Patient was directed to general surgery clinic for his liver cyst. Postoperative chest X-ray was normal in postoperative 8. month control (Fig. 3).

## 3 Discussion

Hydatid disease is an endemic disease provoked by Echinococcus granulosus especially in countries where farming is an important source of income like Turkey, India, Africa and middle Eastern countries [2]. The most common symptoms reported are: chest pain, cough, dyspnea and hemoptysis (3). The most significant complication is cyst rupture; cyst material may drain either into bronchia resulting with chest pain, cough, hemoptysis, or vomica; or into the pleural cavity, causing pneumothorax, pleural effusion, or empyema. Another considerable complication is the infection of the cyst, presenting with pulmonary abscess [3].

Preoperative diagnosis of hydatid cyst is assured with clinical, radiological and serological outcomes. In our patient, history of expectoration of watery material, a thick walled giant cystic lesion seen in Chest X-ray, a cavitary giant cystic lesion determined in Thorax CT oriented us to consider pulmonary hydatid cyst. Perioperative diagnosis was promoted with macroscopic outlook of the cyst and germinative membrane.

Radiologic survey is the basic step in preoperative diagnosis of pulmonary hydatid cysts. In chest X-Ray, cysts are well defined circular mass. Floating membranes inside the cyst are seen with the rupture of the cyst, so called 'water-lily' or 'meniscus sign' [4].

Thorax CT is especially helpful in complicated cysts cases, for instance to illustrate a cyst wall contour deformity in a ruptured cyst. Infected cysts present in CT as poorly defined masses with high internal density and contrast enhancement around the cyst wall with the injection of contrast material [3]. In our patient, there was a giant 130*110 mm well contoured lobulated cavitary lesion detected in the right middle lobe of the lung without finding of rupture. However, history of hydatid vomica directed us to think parenchymatous reaction to bronchial rupture [4]. Ultrasonogram is especially functional for diagnosis when daughter cysts and hydatid sand is exhibited [4].

The most common location of hydatid cysts are lungs and liver; heart, brain, bone and muscle are rarely affected [5]. Our patient had a concomitant Grade II hydatid cyst with round protruding counters in the right liver lobe. Serologic tests, most commonly IgG enzyme linked immunoassay (ELISA) and indirect hemagglutination can also be used to sustain the diagnosis, Nevertheless, we couldn't investigate serology due to the laboratory conditions [6].

Pulmonary hydatid cysts are generally treated with surgery. Surgical procedures purpose removal of whole cyst while protecting the lung parenchyma and avoiding intraoperative spreading. Surgical techniques comprise pericystectomy, enucleation, cystostomy with capitonnage, open aspiration, and lung resection. Enucleation comprises removal of the cyst with germinative membrane. It is proper for small pulmonary hydatid cysts <5 cm [7]. With enucleation, pericyst is left; so, there is a risk of postoperative air leak and infection. Pericystectomy includes removal of hydatid cyst together with the pericyst [7].

Cystostomy and capitonnage is the most commonly used treatment modality of hydatid cyst. Cystotomy comprises of aspiration of the cystic fluid and removal of the germinative membrane. Capitonnage derogates the risk of the infection of the residual cavity, airway leak, and empyema, but there is a risk of corruption of the lung parenchyma [7]. Our patient was also treated with cystostomy with capitonnage successfully.

Other surgical methods include cystostomy with the repair of the bronchial leakages alone [7]. Pneumonectomy, segmentectomy should be performed to cysts involving whole hemithorax or the whole segment respectively; and lobectomy should be performed only in large abscessed cysts [3].

Antihelminthic therapy is used as adjunct therapy in postoperative medication in recurrent disease or in patients with multiple cysts. Postoperative medication of our patient was ensured with albendazole successfully [8].

## 4 Conclusion

Surgery is the treatment regimen for pulmonary hydatid cysts and antihelmintic therapy is adviced to eliminate recurrences postoperatively.

## Ethical approval

The ethical approval has been exempted as it was not necessary in this case report by our institution.

## Sources of funding

This research received no specific grant from any funding agency in the public, commercial, or not-for-profit sectors.

## Author contribution

TA gathered the patient's data and wrote the manuscript. TA, CB participated in the surgery. TA, CB reviewed manuscript. All authors approved the final manuscript.

## Conflicts of interest

Non declared.

## Research registration number

Registration of our study at http://www.researchregistry.com is waived because it registers case series or other group studies or first in man cases, our case is a single patient which is not a first in man.

## Guarantor

Tuba Apaydın,MD.

## Provenance and peer review

Not commissioned, externally peer-reviewed.

## Appendix A Supplementary data

SCARE Guideline Checklist SCARE Guideline Checklist
